# Sero-diagnosis of Active *Mycobacterium tuberculosis* Disease among HIV Co-infected Persons using Thymidylate Kinase based Antigen and Antibody Capture Enzyme Immuno-Assays

**DOI:** 10.4172/2161-1068.1000241

**Published:** 2017-05-31

**Authors:** Misaki Wayengera, Ivan Mwebaza, Johnson Welishe, Cynthia Nakimuli, David P Kateete, Eddie Wampande, Samuel Kirimunda, Lois Bayigga, Carol Musubika, Peace Babirye, Benon Asiimwe, Moses L Joloba

**Affiliations:** 1 Department of Pathology, Unit of Genetics and Genomics, School of Biomedical Science, Makerere University College of Health Sciences, Kampala, Uganda; 2 Department of Immunology/Molecular Biology/Mycobacteriology, School of Biomedical Sciences, Makerere University College of Health Sciences, Uganda; 3 Department of Medical Microbiology, School of Biomedical Sciences, Makerere University College of Health Sciences, Kampala, Uganda

**Keywords:** Tuberculosis, Immunodiagnosis, *Mycobacterium tuberculosis*, Thymidylate kinase, Antigen, Antibody, Enzyme immuno-assays

## Abstract

**Background:**

Clinical and laboratory diagnosis of Active Tuberculosis (ATB) and latent *Mycobacterium Tuberculosis* (*M. tuberculosis*) infections (LTBI) among people living with HIV/AIDS (PLWHA) presents formidable challenges. In the past, WHO issued an advisory against the use of existing TB sero-diagnostics. Emerging evidence, however, points to a precision of TB sero-diagnostics based on secretory rather than structural *M. tuberculosis* antigens. We hypothesized that secretory levels of *M. tuberculosis* thymidylate kinase (TMKmt) can Designate ATBI from LTBI and no TB (NTB). Here, we report in-house validation studies of levels of TMKmt antigen (Ag) and host specific TMKmt antibody (Ab) amongst HIV +ve and HIV −ve participants.

**Methods and Results:**

Direct TMKmt Ag and host specific IgG Ab detection EIAs were conducted on broadly consented, stored serum (N=281[Ag] *vs*. 214 [Ab] respective) samples stratified as either HIV +ve or HIV−ve ATB relative to LTBI and No TB. On one hand, UG-peptide 1 and its PAb-based EIAs accurately diagnosed ATB relative to LTBI and NTB among HIV +ve subjects {irrespectively: (a) Ag detection ATB=OD>0.490; 95% CI: 0.7446 to 0.8715 *vs*. LTBI=OD<0.490; 95% CI 0.4325 to 0.4829 *vs*. NTB=OD<0.26; 95% CI 0.1675 to 0.2567 and (b) TMKmt specific IgG detection ATB=OD>1.00; 95% CI 1.170 to 1.528 [HIV +ve] and 2.044 to 2.978 [HIV −ve] respectively *vs*. LTBI=OD<1.00; 95% CI 0.2690 to 0.6396 *vs*. NTB=OD<; 95% CI 0.1527 to 0.8751}. HIV −ve ATB presented with Ag levels greater than NTB and less than LTBI (i.e. ATB −ve=<0.490 ODs>0.26), but displayed better ant-TMKmt IgG responses (OD>2.00; 95% CI 2.044 to 2.978) relative to HIV +ve ATB (OD<1.600; 95% CI 1.170 to 1.528); suggesting a better control of *M. tuberculosis*-septicemia. On the other hand, UG-peptide 2 and its PAb-based EIAs did not demonstrate ATB diagnostic potential regardless of HIV sero-status, except towards designating NTB.

**Conclusions:**

TMKmt Ab and Ag detecting EIAs based on UG-peptide 1 and its derivative PAb can accurately demarcate ATB from LTBI and NTB among HIV +ve subjects.

## Introduction

Tuberculosis (TB) is a leading infectious co-morbidity among people living with HIV/AIDS (PLWHA). TB is caused by infection with *Mycobacterium tuberculosis* (*M. tuberculosis*) [1,2]. Infection with *M. tuberculosis* may either be asymptomatic (latent *M. tuberculosis* infection, LTBI) or symptomatic (active TB, ATB or simply TB) [2]. A group of either not exposed (the case for citizens of TB-low burden countries) or persistently exposed yet not infected persons within TB high-burden settings (no TB, NTB) is known to exist [1,2]. HIV co-infection results in more severe and disseminated TB disease.

Clinical and laboratory diagnosis of TB among PLWHA presents formidable challenges [1,2]. HIV co-infection among people with LTBI increases the risk of developing ATB from 10% over a lifetime to 10% per year. In as much as the use of either highly active antiretroviral therapy (HAART) or Six to nine months of isoniazid (INH) therapy reduces this increased risk for developing active TB, the risk remains elevated after a good response to either regimen. Moreover, due to absence of diagnostics that accurately designate those at high-risk of reactivation, INH therapy cannot be effectively targeted [1,2].

Overall, precise and reliable demarcation of *M. tuberculosis* infection status is relevant both towards individualized case-management and public health control of TB [2–5]. Designation of *M. tuberculosis* infection status, however, remains a global challenge [2–7]. On one hand, the vast reservoir of LTBI from which ATB accrue remains largely undetectable due to absence of cheap, easy to use, rapid point of care diagnostics (POC) for the same [5,7]. New assays that are predictive of high-risk (or incipient) are needed to enable institution of chemoprohylactic measures and curtail the build-up of symptomatic TB [5–7].

Diagnosis of LTBI was traditionally premised on the Tuberculin Skin Test (TST: also known as the purified protein derivative or simply PPD, skin test) [3,8–12]. The TST is nevertheless unreliable in presence of HIV co-infection, a common occurrence within TB high burden settings of sub-Saharan Africa [3,5–7]. As a result, several TB prognostic assays based on detecting interferon gamma responses (and are thereby denoted interferon gamma release assays or simply IGRAs) have emerged [3,8–12]. IGRAs, however, lack the inexpensive platforms to allow routine application within resource constrained settings and cannot accurately designate high-risk LTBI [3]. On the other hand, early detection of symptomatic or ATB remains a frequent problem within many TB high burden nations [5–12].

Most centers where symptomatic TB patients first come in contact with primary health providers lack the laboratory setup for detecting TB disease leading to significant drops in case-detection (and ultimately-treatment) rates of AT [8–12]. These two challenges emphasize the need for new (or modified) biomarkers which are effective for delineating *M. tuberculosis* infection status and can be mount onto existing inexpensive platforms widely used for infectious disease diagnosis within resource limited settings such as lateral flow immunochromatographic strips [13].

The World Health Organization (WHO) has in the past issued a negative recommendation against the use of existing TB sero-diagnostics, advocating for research and development (R&D) of newer serologic biomarkers for TB [4–7]. A critical review of related literature, however, reveals that majority tested serologic biomarkers so far, were derived from structural *M. tuberculosis* antigens whose antisera levels do not depend on the physiological properties of *M. tuberculosis* that assail the pathogenesis of TB disease.

Wassie et al. [14] have shown that humoral responses specific against both structural and secretory *M. tuberculosis* antigens are mounted by the host during the course of infection. Insight from the study of host humoral responses against *M. tuberculosis* Lipolytic enzymes by Brust et al. [15], however, precisely suggests that only those humoral responses against antigens whose secretory levels depend on particular aspects of the physiology of *M. tuberculosis* offer the best candidate biomarkers for immunodiagnosis of TB. Previous studies using *in-vivo* expression technologies suggest that bacterial pathogenicity depends on genes essential for bacterial growth and intracellular survival [16]. Growth and proliferation of *M. tuberculosis* is known to be arrested or very slow during LTBI (a state of dormancy) and set-lose in ATB [1–3].

Growth and proliferation of *M. tuberculosis* therefore presents a potential surrogate marker for TB disease progression. A noticeable gap, nonetheless, is the absence of easy to use and culture-indepedent assays for detecting *M. tuberculosis* growth and proliferation. *M. tuberculosis* thymidylate (a.k.a thymidine monophosphate, TMP) kinase or simply TMKmt is a phosphotransferase that catalyses the phosphorylation of deoxythymidine monophosphate (dTMP) to the diphosphate precursor used to generate deoxythymidine-triphosphate (dTTP) that finally gets integrated into the growing *M. tuberculosis* DNA chain [17–21].

Secretory levels of TMKmt are characterized by a 10–20-fold increase after the G1/S transition, and remain high until about the time of cell division when they then decline rapidly. While groups elsewhere are researching TMKmt as a potential target for TB drug development, only our group has previously proposed secretory levels of TMKmt as a biomarker for *M. tuberculosis* growth and proliferation both among *in-vitro* and *in-vivo* cultures [17,21].

We have in the past identified 2 novel *M. tuberculosis* thymidylate kinase (TMKmt) epitopes whose capability to designate TB exposure status among a largely HIV −ve cohort by host-specific antibody (Ab: IgM and IgG) detecting enzyme immuno-assays (EIA) was reported (unpublished data) [22,23]. In this study, we aimed to expand our validaton studies to include large populations of HIV +ve and HIV −ve participants alongside Ag detection EIAs.

## Materials and Methods

### Pre-amble to methodology

The identity of the two TMKmt epitopes referenced in this study has been described elsewhere [23,24]. Briefly, (i) using the entire 214 amino acids sequences of TMKmt (SP “|O05891|”) and five biophysical profiles (accessibility, antigenicity, beta-turn, flexibility, and hydophilicity) in the immune epitope database analysis resource (IEDB-AR), the 27 AA long 148_ERSRGRAQRDPGRARDNYERDAELQQR peptide was predicted to be the best continuous linear B cell epitope by all profiles (4/5, 80%) except antigenicity [25–29].

Second, using the crystal structure of TMKmt (PdB entry: “1g3u”) and discontinuous B cell epitope software DiscoTope, we derived 22 amino acids (A:G57, A:E148, A:S150, A:R151, A:G152, A:R153, A:A154, A:Q155, A:R156, A:D157, A:P158, A:G159, A:A160, A:A161, A:R162, A:A163, A:N164, A:E166, A:R167, A:D168, A:A169, A:T179) as the best discontinuous or non-linear epitope [27]. Both peptide-epitope sequences were cross validated by BLAST across microbialand conserved domain databases (CDD) [29,30].

The referenced two synthetic TMKmt-peptide-epitopes and their derivative polyclonal antibodies (PAbs, GeneCUST, Luxemburg) were used to detected TMKmt host specific serum antibody (Ab, IgG) and TMKmt Antigen (Ag) levels by direct enzyme immuno-assays (EIA) based on HRP-labelled goat anti-human or anti-rabbit specific secondary(IgG), respectively as is further decribed in the design and methodology section. All clinical samples used were designated as either LTBI/NTB or ATB [all pulmonary TB (PTB)] using Quantiferon^®^ gold IGRA assay and acid fast bacillus (AFB) smear microscopy/culture, respectively.

### TMKmt Antigen Detection Enzyme Immuno-Assays (Ag-EIA)

#### Design: Cross sectional laboratory study

##### Materials and supplies

Rabbit derived polyclonal antibodies (PAb-0655 and PAb-0656 respectively) against the 2 TMKmt peptide-epitopes (GeneCUST, Luxemburg), plain ELISA plates (flat bottom, Nunc), Bovine Serum Albumin (BSA, In-vitrogen, USA), goat anti-rabbit IgG (HRP labbelled, Bio-Rad, France), Phosphate Buffered Saline (PBS), and the enzymatic substrate tetramethylbenzidine (TMB). Broadly consented, stored serum ([N=281 for Ag detection]) samples stratified as HIV +ve (139) or HIV −ve (47) ATB relative to LTBI (83) and No TB (10). A dilute (0.01 ng/ul) Purified recombinant TMKmt cloned, expressed and purified from *E. coli* (See supporting file 2), was used as positive control. All experiments were run in duplicates.

##### Interventions

For detection of TMKmt Ag in participant serum (i) 1ul of serum was dissolved in 1000 µl or 1 ml of freshly prepared phosphate buffered saline. (ii) 100 µl of resultant serum-diluent was then pipetted into each of the wells of a sterile 96-well microtiter plate (Nunc) and the plate incubated overnight. (iii) The plated wells were then blocked once the following day using 5% BSA in PBS and incubated at 37°C for 30 mins, after which excess solution was discarded and plate left to dry.

Blank wells were also made, by adding only PBS rather than sample. The wells-in-use were then washed with PBS three times using an automated plate-washer. (iv) TMKmt specific rabbit polyclonals (either PAb-0655 or PAb-0656) was added and plates incubated at 37°C for 30 mins, after which excess solution was discarded and plate left to dry. The wells-in-use were then washed with PBS three times using an automated plate-washer. (v) 100 µls of goat anti-rabbit IgG horse-raddish peroxidate conjugate was added, and the plates incubated at 37°C for another 1 hour.

During this incubation, the enzyme substrate was prepared by adding 1 volume of substrate (TMB) to 1 volume of diluent (hydrogen peroxide) in volumes enough for all thewells in use. (vi) 200 µl of freshly prepared substrate was added to each well (purple-bluish color developed in all except A-BX1 blank wells). (vii) The reaction was stopped by adding 100 µl of dilute (1mol/L) H_2_SO_4_. The intensity of the reaction in each well was henceafter determined by reading the plate at an optical density (OD) of 450 nm using a single filter of an automated ELISA plate reader (PR 3100, Bio-Rad).

##### Measured variables

Levels of TMKmt Ag in study serum or blanks was qualitatively detected as a function of the OD of each well.

##### Treatment of results

Raw data was cleaned by substrating ODs of blanks from those of test wells. The issuing adjusted ODs were averaged across the duplicate runs for each test well. Resultant average adjusted ODs were analysed by both PRISM software, and Excel. Graphs were also drawn by GraphPad. For each OD read (essentially done in triplicate), a 95% Confidence interval (CI) read was computed, alongside the slopes and P-values. Excel sheets were used for correction of average sample OD readings by substracting OD reading of the blank wells. Only CIs were considered as this exploratory study aimed to demonstrate the accuracy and reliability (replicability) of these assays, prior to undertaking planned future evaluations for sensitivity, specificity, positive and negative predictive values (PPV and NP as well as receiver operator characteristcs (ROCs) curves. To strengthen replicability, testing was done in separate groups of each TB-status category and results analyzed and presented separate clusters of the same group.

### TMKmt Host Specific Antibody (IgG) Enzyme Immuno-Assays (Ab-EIA)

#### Design: Cross sectional laboratory study

##### Materials and supplies

Synthetic homologues of 2 TMKmt peptide-epitopes (GeneCUST, Luxemburg), plain ELISA plates (flat bottom, Nunc), Bovine Serum Albumin (BSA, In-vitrogen, USA), goat anti human IgG (HRP labbelled, Bio-Rad, France), Phosphate Buffered Saline (PBS), and the enzymatic substrate tetramethylbenzidine (TMB). Broadly consented, stored serum ([N=214 for Ab detection]) samples stratified as HIV +ve (122) or HIV −ve (37) ATBI relative to LTBI (47) and No TB (8). All experimengts were run in duplicates.

##### Interventions

For detection of host TMKmt specific IgG humoral responses against TMKmt, we (i) dissolved 1ug (conc: 10 mg/ml) of individual synthetic peptide by adding 100 µl of freshly prepared phosphate buffered saline (PBS was prepared by dissolving ¼ of a 250 mg tablet in 50ml PCR grade water). (ii) 100 µl (0.001 ng) of individual synthetic peptide (UG-Peptide-01 and UG-Peptide-02) was then pipetted into each of the wells of a sterile 96-well microtiter plate (Nunc) and the plate incubated overnight. (iii) The plated wells were then blocked once the following day using 5% BSA in PBS and incubated at 37°C for 30 mins, after which excess solution was discarded and plate left to dry. (iv) 100 µl of PBS was added to each assigned wells, followed by addition of 10 µl (1:100 dilution) of samples into the respective wells; after which the plate was shaken at 15 HZ for 16 secs, and incubated for 1 hour at 37°C. Blank wells were also made, by adding only PBS rather than sample.

The wells-in-use were then washed with PBS three times using an automated plate-washer. (v) 100µls of goat anti-human IgG horse-raddish peroxidate conjugate was added, and the plates incubated at 37°C for another 1 hour. During this incubation, the enzyme substrate was prepared by adding 1 volume of substrate (TMB) to 1 volume of diluent (hydrogen peroxide) in volumes enough for all thewells in use. (vi) 200 µl of freshly prepared substrate was added to each well (purple-bluish color developed in all except A-BX1 blank wells). (vii) The reaction was stopped by adding 100 µl of dilute (1 mol/L) H_2_SO_4_. The intensity of the reaction in each well was henceafter determined by reading the plate at an optical density (OD) of 450nm using a single filter of an automated ELISA plate reader (PR 3100, Bio-Rad).

##### Measured variables

Levels of TMKmt specific humoral (IgG) responses in study serum or blanks was qualitatively detected as a function of the OD of each well.

##### Treatment of results

Raw data was cleaned by substrating ODs of blanks from those of test wells. The issuing adjusted ODs were averaged across the duplicate runs for each test well. Resultant average adjusted ODs were analysed by both PRISM software, and Excel. Graphs were also drawn by GraphPad. For each OD read (essentially done in triplicate), a 95% Confidence interval (Cis) read was computed, alongside the slopes and P-values. Excel sheets were used for correction of average sample OD readings by substracting OD reading of the blank wells.

Only CIs were considered as this exploratory study aimed to demonstrate the accuracy and reliability (replicability) of these assays, prior to undertaking planned future evaluations for sensitivity, specificity, positive and negative predictive values (PPV and NP as well as receiver operator characteristcs (ROCs) curves. To strengthen replicability, testing was done in separate groups of each TB-status category and results analyzed and presented separate clusters of the same group.

## Results

Direct TMKmt Ag and specific IgG Ab Detection EIA were conducted on broadly consented, stored serum ([N=281 for Ag detection] *vs*. [N=214 for Ab detection]) samples stratified as either HIV +ve (139 *vs*. 122) or HIV −ve (47 *vs*. 37 for Ag and Ab detection respectively) ATB relative to LTBI (83 *vs*. 47) and No TB (10 *vs*. 8).

### Ug-peptide 1 based antigen and antibody detection enzyme immuno-assays

In general, both UG-peptide 1 Ag and Ab EIAs accurately diagnosed ATB relative to LTBI and NTB among HIV +ve subjects. On one hand, TMKmt Ag detection using polyclonal (Pab-0655) derived from this eiptope revealed adjusted optical densities (ODs) among HIV +ve ATB participant-serum greater than (>) 0.490, best fit 0.8081 ± 0.0329; 95% CI: 0.7446 to 0.8715. This was above the observed TMKmt Ag levels among LTBI participant-serum, all of whose ODs were less than (<) 0.490, best fit 0.4577 ± 0.0129; 95% CI 0.4325 to 0.4829.

Further still, TMKmt Ag levels among both ATB and LTBI participant-serum were significantly greater than readings observed among NTB participant-serum, all of which were less than (<) 0.26, best fit 0.2121 ± 0.0213; 95% CI 0.1675 to 0.2567.

**Figure 1:**
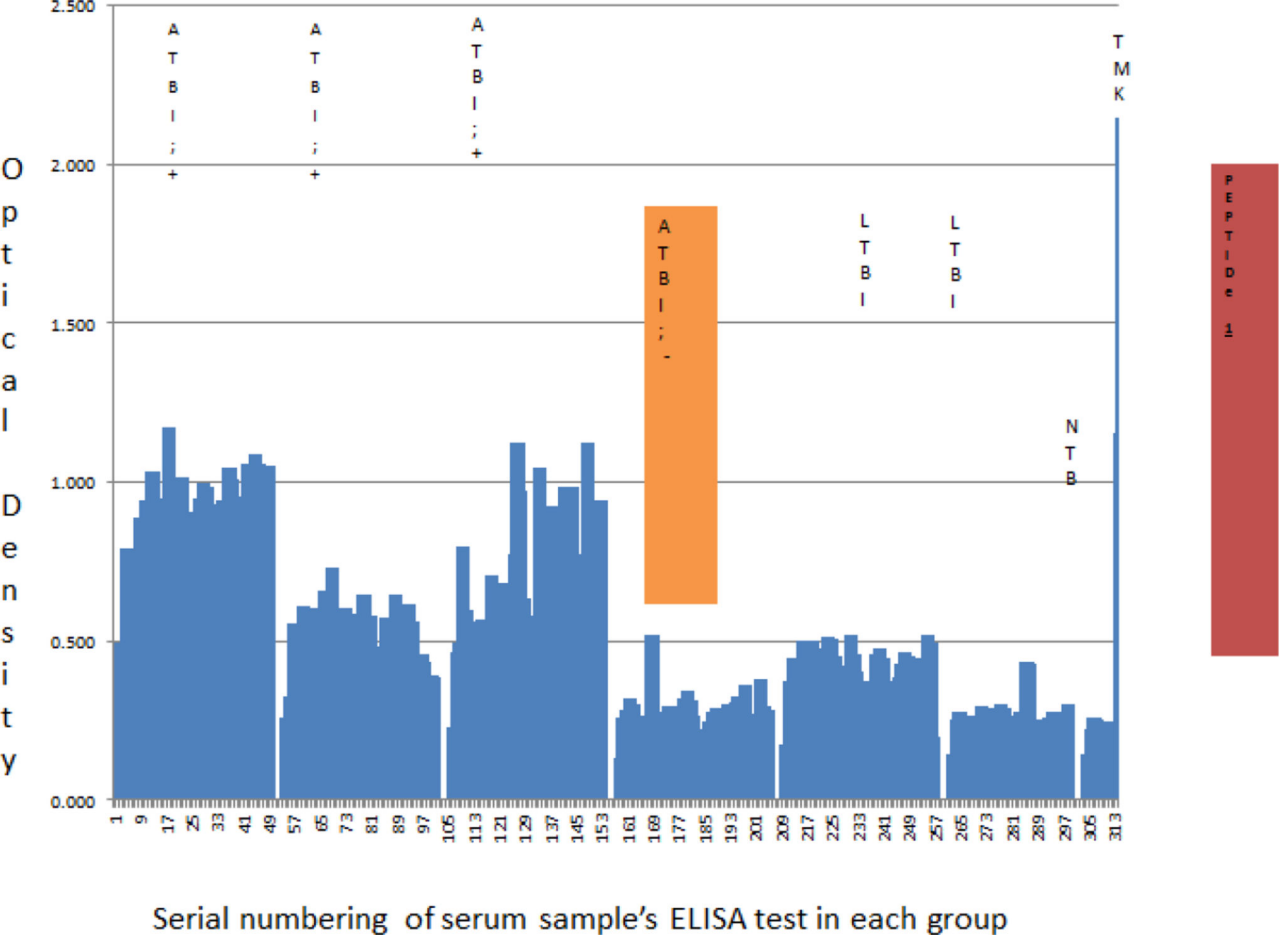
TMKmt Ag levels detected by UG-peptide 1 derivative PAb-0655 EIAs among sera of HIV+ and HIV −ve participants with ATB, LTBI, and NTB. This figures shows levels of TMKmt Ag detected by UG-peptide 1 derivative PAb-0655 EIAs among 281 serum samples stratified as either HIV +ve (139) or HIV −ve (47) ATB versus to LTBI (83) and No TB (10). TMKmt Ag capture in serum can differentiate between active and latent TB among sero-postives (ATB=OD>0.490; LTBI=OD<0.490); but sero-negatives control ATB presents with similar Ag levels as LTBI (i.e. OD<0.490). The accurancy and specificity of this Ag detection EIA for TMKmt among HIV +ve participant serum is demonstrated by the relative ODs [best fit 0.1660 ± 0.0112, 95% CI: 0.1179 to 0.2141] observed with a highly dilute solution (0.01 ng/ul) of recombinant TMKmt expressed and purified from within *E. coli*. *M. tuberculosis* growth and proliferation-which actively occurs in the blood of PLWHA due to the dessiminated nature of TB disease seen, is ably and precisely demonstrated by TMKmt Ag capture EIAs based on UG-peptide 1. HIV −ve persons have less circulating TMKmt Ag (representative of better control of *M. tuberculosis* infection i.e. ODs<0.300; as is LTBI and NTB) relative to HIV +ve (ODs>0.300). The immunodeficiency that assails HIV infection or AIDS is assailed by dessimnated TB disease that makes blood a mileu for *M. tuberculosis* growth and proliferation *in-vivo*, permitting precise detection of its growth and proliferation depedant antigens (TMKmt) in serum.

TMKmt Ag levels among serum of HIV −ve participants with ATB displayed, values greater than TMKmt Ag detected among serum of participants with NTB but less than TMKmt Ag among LTBI participant serum [best fit 0.2557 ± 0.0182; 95% CI: 0.2195 to 0.2918] (Figure 1, Supporting Figure 1, Table 1 and Supporting file 1 section A).

The accuracy and specificity of this Ag detection EIA for TMKmt among HIV+ve participant serum is demonstrated by the relative ODs [best fit 0.1660 ± 0.0112, 95% CI: 0.1179 to 0.2141] observed with a highly dilute solution (0.01 ng/ul) of recombinant TMKmt expressed and purified from within *E. coli* (Supporting file 2). Moreover, the high levels of TMKmt Ag observed in serum of HIV+ve participants with ATB relative to their HIV −ve counterparts with the same diagnosis, is consistent with the immuno-pathogenesis of TB among PLWHA. Specifically, among PLWHA, TB disease assumes a largely dessiminated form wherein a septicemia comprising *M. tuberculosis* bacilli is seen [1–10]. The immunodeficiency that assails HIV infection or AIDS is assailed by dessimnated TB disease that makes blood a mileu for *M. tuberculosis* growth and proliferation *in-vivo*, permitting precise detection of its growth and proliferation dependant antigens (TMKmt) in serum. Similar findings can therefore be expected among bodily fluids were active *M. tuberculosis* growth and proliferation occurs per TB disease site, such as sputum or pleural fluid for pulmonary TB (PTB), ascites for abdominal TB, and cerebral spinal fluid (CSF) for TB meningites (TBM).

**Figure 2:**
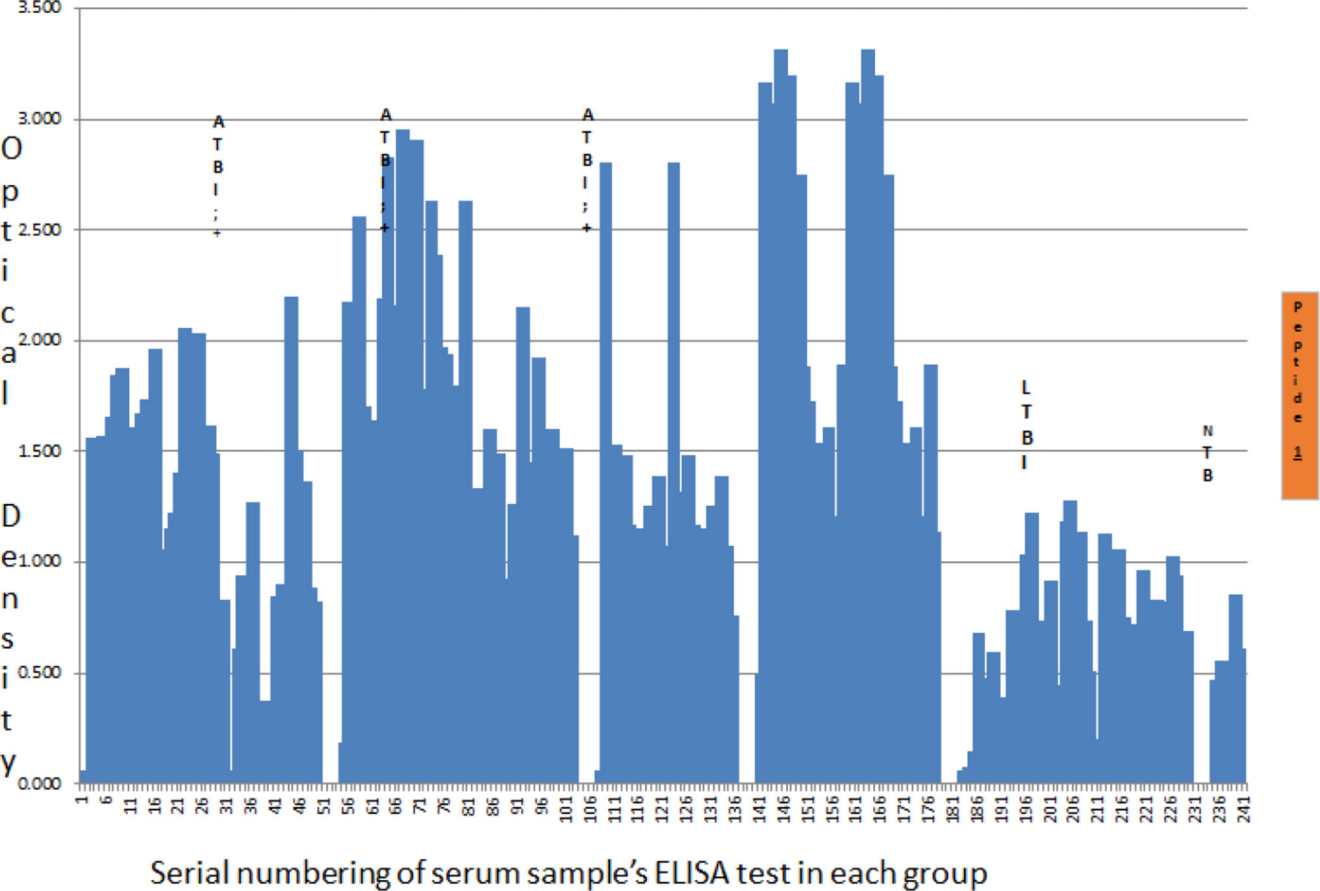
Host TMKmt specific IgG levels detected by UG-peptide 1 based EIAs among sera of HIV+ and HIV −ve participants with ATB, LTBI, and NTB. This figures shows levels of anti-TMKmt specific IgG responses detected by UG-peptide 1 based EIAs among 214 serum samples stratified as either HIV +ve (122) or HIV−ve (37) ATB versus LTBI (47) and No TB (8). Host TMKmt specific IgG levels detected by UG-Peptide 1 EIA capable of differentiating ATB from LTBI (ATBI=OD>1.00 *vs*. LTBI=OD<1.00); unlike peptide 2. HIV −ve persons display better anti-TMKmt IgG responses (ODs>1.500) relative to HIV +ve (ODs<1.500). This data is consistent with Ag capture results that reveal better *M. tuberculosis* control among HIV −ve persons relative to HIV +ve. The notable low levels of anti-TMKmt specific IgG Ab responses among PLWHA relative to their HIV −ve counter-parts tallies with the immunodeficiency seen in the former group. This makes UG-peptide 1 and its derivative PAb-0655 potential correlates of immune function among PLWHA.

Therefore, despite the earlier noted inadequacy of sero-diagnostics for the purpose of TB detection, two major alterations in the design and testing of our assays explain these precise findings i.e. that (i) the *M. tuberculosis* antigen targeted is a secretory predictive biomarker of *M. tuberculosis* growth and proliferation, and (ii) TB disease among PLWHA is dessiminated, making serum a mileu for *M. tuberculosis* growth and proliferation [4–7,15]. On the other hand, TMKmt specific host-IgG detection displayed an inverse but consistent picture to that of Ag capture. Specifically, Ab-levels among ATB donor serum irrespective of HIV serostatus were=OD>1.00 [95% CI 1.170 to 1.528 among HIV +ve participants relative to and 2.044 to 2.978 for HIV −ve participants]. This, when compared to Ab-depicting ODs observed among serum of LTBI and NTB participants regardless of HIV serostatus, which were lower than (<) 1.00 [LTBI 95% CI: 0.2690 to 0.6396] *vs*. [NTB 95% CI: 0.1527 to 0.8751]. HIV −ve ATB presented with similar Ag levels as LTBI (i.e. OD <0.490), but displayed better ant-TMKmt IgG responses (OD>2.00; 95% CI 2.044 to 2.978) relative to HIV +ve ATB (OD<1.600; 95% CI 1.170 to 1.528); suggesting a better control of *M. tuberculosis*-septicemia (Figure 2, Supporting Figure 2, Table 2, and Supporting file S1 section B).

Moreover, the notable low level of anti-TMKmt specific IgG Ab responses among PLWHA relative to their HIV −ve counter-parts tallies with the immunodeficiency seen in the former group. In as much as variation in human leucocyte antigen (HLA) phenotypes have been reported to impact on IgA, IgM and IgG responses to *M. tuberculosis* culture filtrate and the 30 KDa antigen, these had little or no impact on the overall Ab differences in serum of participants with HIV+ and HIV −ve ATB [22]. This makes UG-peptide 1 and its derivative PAb-655 potential correlates of immune function among PLWHA [1–3].

### Ug-peptide 2 based antigen and antibody detection enzyme immuno-assays

Unlike the inverse but concordant Ag and Ab precision for delineating ATB from LTBI and NTB reported above for UG-peptide 1 and its derivative PAB-0655, UG-peptide 2 and its PAb-based EIAs did not demonstrate specific ATBI diagnostic potential regardless of HIV sero-status.

**Figure 3:**
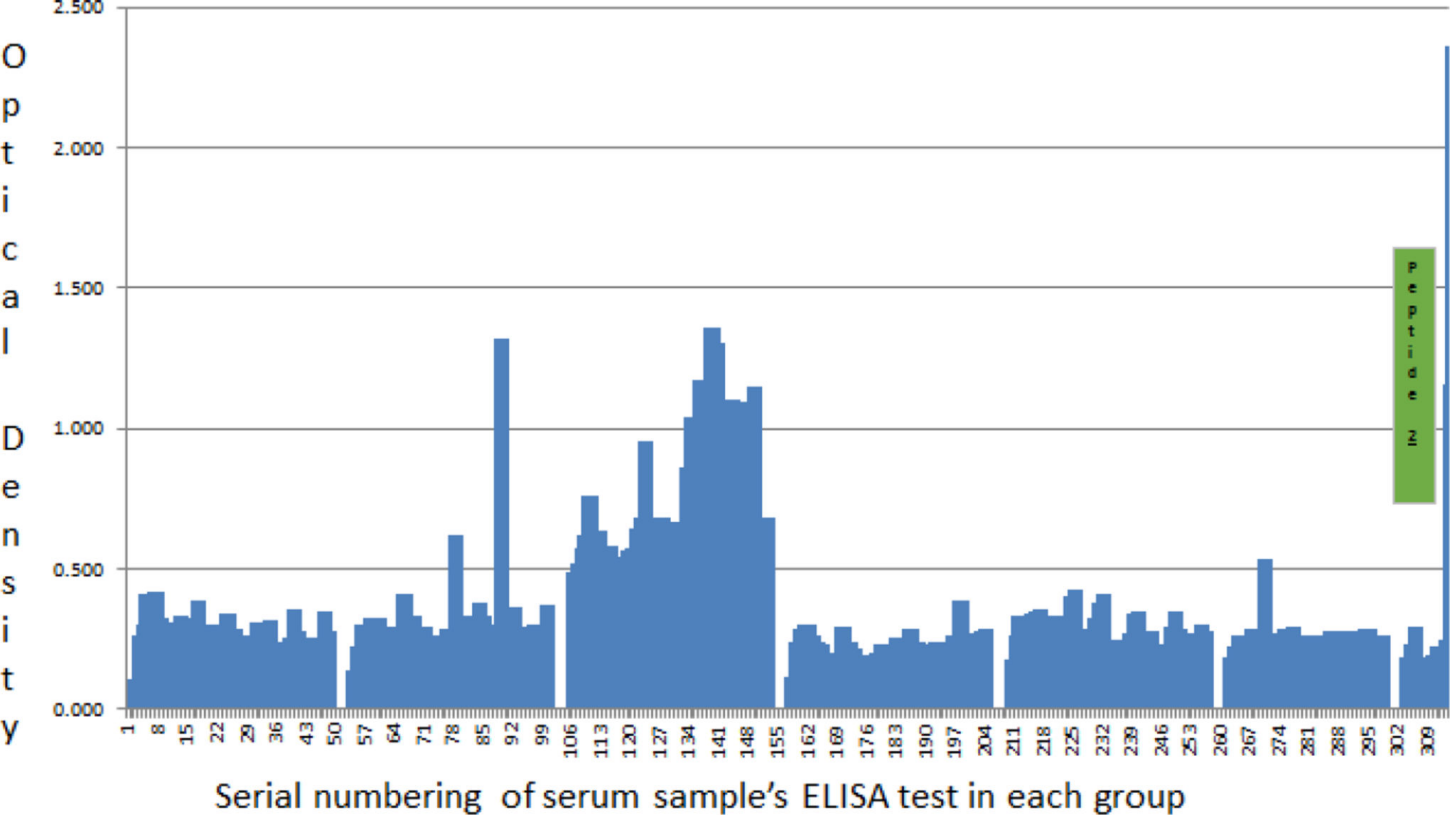
TMKmt Ag levels detected by UG-peptide 2 derivative PAb-0656 EIAs among sera of HIV+ and HIV −ve participants with ATB, LTBI, and NTB. This figures shows levels of TMKmt Ag detected by UG-peptide 2 derivative PAb-0656 EIAs among 281 serum samples stratified as either HIV +ve (139) or HIV −ve (47) ATB versus to LTBI (83) and No TB (10). PAb-0656 (which is derived from UG-peptide 2) detected low levels of TMKmt Ag among 2 of 3 stratifications of HIV +ve ATB participant-serum less than (<) 0.160 [best fit 0.1039 ± 0.0275; 95% CI: 0.0500 to 0.1578]. This when compared to Ag levels detected by the same PAb among participant serum of HIV −ve ATB and LTBI (regardless of serostatus), all of which were above (>) 0.160 [HIV −ve ATBI, best fit 0.2095 ± 0.0110; 95% CI: 0.1876 to 0.2313 and LTBI, best fit 0.2994 ± 0.0109; 95% CI: 0.2780 to 0.3208]. This when compared to Ag levels detected by the same PAb among participant serum of HIV - ve ATB and LTBI (regardless of sero-status), all of which were above (>) 0.160 [HIV −ve ATB, best fit 0.2095 ± 0.0110; 95% CI: 0.1876 to 0.2313 and LTBI, best fit 0.2994 ± 0.0109; 95% CI: 0.2780 to 0.3208]. Unlike the case with PAb-0655, there was no clearly demonstrated specificity for the same dilution (0.01ng/uL) of recombinant TMKmt expressed in E. coli [best fit −0.0430 ± 0.0335; 95% CI: −0.1873 to 0.1013], suggesting Ag detection results based on PAb-0656 may in part be due to non-specific bindiing. Overall, levels of TMKmt Ag detected among HIV +ve ATB were not different from LTBI but were evidently greater than that detected than NTB (OD>0.250). TMKmt Ag levels detected by UG-peptide 2 derivative PAb-0656 are unable to differentiate ATB from LTBI.

On one hand, contrary to the high levels of TMKmt Ag detected among HIV +ve ATB participant serum using PAb-0655(derived from UG-peptide 1) and potentially explained by poor control of *M. tuberculosis* septicemia, PAb-0656 (which is derived from UG-peptide 2) revealed low levels of TMKmt Ag among 2 of 3 stratifications of HIV +ve ATB participant-serum less than (<) 0.160 [best fit 0.1039 ± 0.0275; 95% CI: 0.0500 to 0.1578]. This when compared to Ag levels detected by the same PAb among participant serum of HIV −ve ATB and LTBI (regardless of sero-status), all of which were above (>) 0.160 [HIV −ve ATB, best fit 0.2095 ± 0.0110; 95% CI: 0.1876 to 0.2313 and LTBI, best fit 0.2994 ± 0.0109; 95% CI: 0.2780 to 0.3208]. Furthermore, unlike the case with PAb-0655, there was no clearly demonstrated specificity for the same dilution (0.01 ng/ul) of recombinant TMKmt expressed in *E. coli* [best fit −0.0430 ± 0.0335; 95% CI: −0.1873 to 0.1013], suggesting Ag detection results based on PAb-0656 may in part be due to non-specific binding. This is further compounded by the similarly high Ag levels detected among serum of participants designated as NTB [best fit 0.2206 ± 0.0361; 95% CI: 0.1448 to 0.2965] lying in the range of TMKmt Ag that were visibly detected by the same PAb among ATB and LTBI (Figure 3, Supporting Figure 3, Table 3 and Supporting file S1 Section A).

**Figure 4:**
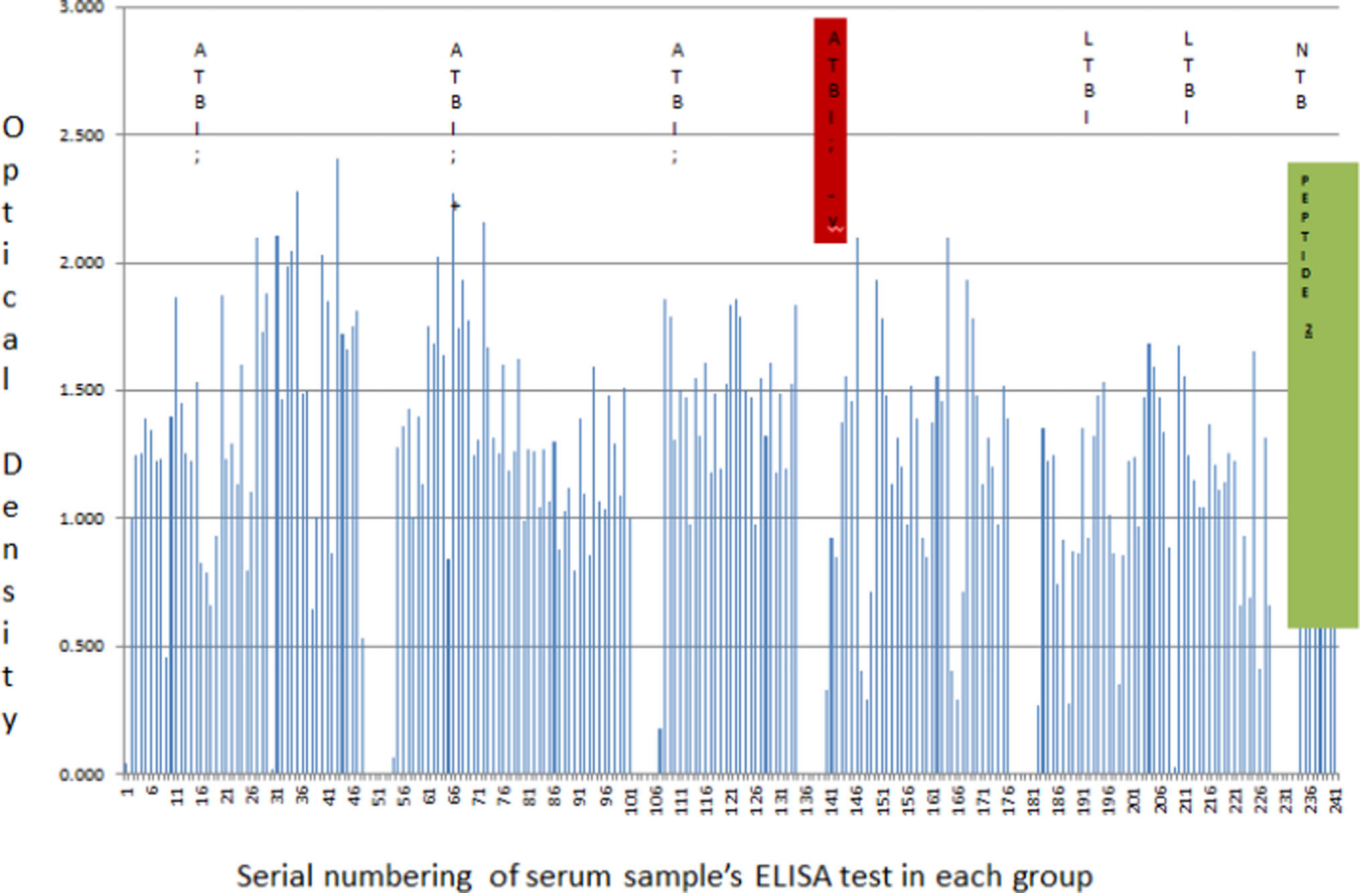
Host TMKmt specific IgG levels detected by UG-peptide 2 based EIAs among sera of HIV+ and HIV −ve participants with ATB, LTBI, and NTB. This figures shows levels of anti-TMKmt specific IgG responses detected by UG-peptide 2 based EIAs among214 serum samples stratified as either HIV +ve (122) or HIV −ve (37) ATB versus LTBI (47) and No TB (8).Due to the potential non-specificity of its derivative PAb, TMKmt specific host-IgG detection by UG-peptide 2 was found to be incapable of differentiating ATB from LTBI. Specifically, other than in 1 of the 3 stratifications of ATB among the HIV sero-positives (who possibly had CD4 functions >500 cells/ml); the rest of Ab levels for both ATB and LTBI regardless of sero-status were found to reside between 0.4300 to 1.45 (i.e. ATB +ve [best fit 1.303 ± 0.07432; 95% CI 1.158 to 1.449] *vs*. ATB −ve [best fit 1.091 ± 0.1794; 95% CI: 0.7330 to 1.449] *vs*. LTBI best fit 1.029 ± 0.09913; 95% CI: 0.8317 to 1.226] *vs*. NTB best fit 0.9316 ± 0.2348; 95% CI: 0.4279 to 1.435]

On the other hand, possibly due to the potential non-specificity of its derivative PAb, TMKmt specific host-IgG detection by UG-peptide 2 was found to be incapable of differentiating ATB from LTBI. Specifically, other than in 1 of the 3 stratifications of ATB among the HIV sero-positives (who possibly had CD4 functions >500 cells/ml); the rest of Ab levels for both ATB and LTBI regardless of sero-status were found to reside between 0.4300 to 1.45 (i.e. ATB +ve [best fit 1.303 ± 0.07432; 95% CI 1.158 to 1.449] *vs*. ATB −ve [best fit 1.091 ± 0.1794; 95% CI: 0.7330 to 1.449] *vs*. LTBI [best fit 1.029 ± 0.09913; 95% CI: 0.8317 to 1.226] *vs*. NTB best fit 0.9316 ± 0.2348; 95% CI: 0.4279 to 1.435] (Figure 4, Supporting Figure 4, Table 4 and Supporting File S1 Section B). The lower values of Ab detected among NTB negative controls, however, suggest that Ab detection based on PAb-656 is not completely useless.

## Discussion

We present here data to support the view that TMKmt Ag and Ab detecting EIAs based on one of our proprietary epitopes and its derivative PAb (denoted UG-peptide 1 and PAb-0655, respectively) can accurately demarcate ATB from LTBI and NTB among HIV +ve subjects (Tables 1 and 2, alongside Figure 1 and 2, respectively). For additional data, see Supporting Figure S1 and S2, and Supporting file S1. This, with reference to purified recombinant TMKmt cloned and expressed in *E. coli* as a positive control and TB negative samples (Supporting Figure S2). In contrast, both Ag and Ab detection EIAs based on UG-peptide 2 and its derivative PAb-0656 were not able to differentiate ATB from LTBI and NTB regardless of HIV serostatus (Tables 3 and 4, alongside Figure 3 and Figure 4). For additional data, supporting Figure S3 and S4, and Supporting file S1).

Specifically, TMKmt Ag capture in serum was found to possses capability to differentiate active from latent *M. tuberculosis* infections among sero-positives (ATB=OD>0.490; LTBI=D<0.490); while sero-negatives ATB presented with similar Ag levels as LTBI (i.e. OD<0.490) (Figure 1 and Table 1). In this respect, we argue that TMKmt Ag capture based on UG-peptide 1 derivative PAb-0655 demonstrated capability to detect higher levels of TMKmt present alongside the myco-septicemia observed among PLWHA co-intected with *M. tuberculosis*. *M. tuberculosis* growth and proliferation-which actively occurs in the blood of PLWHA due to immuno-suppression, is ably and precisely detected by TMKmt Ag capture EIAs based on UG-peptide1. On the contrary, because HIV −ve persons have less circulating TMKmt Ag (inferably representative of better immune control of *M. tuberculosis* infection i.e. ODs<0.300; as is LTBI and NTB) relative to HIV +ve (ODs>0.300); less antigen was detected by PAb-0655. In agreement, host TMKmt specific IgG levels detected by UG-Peptide 1 based EIAs demonstrated ability to differentiate ATB from LTBI (ATB=OD>1.00 *vs*. LTBI=OD<1.00). This implies that HIV −ve persons display better anti-TMKmt specific IgG responses (ODs>1.500) relative to HIV +ve (ODs<1.500). This data is consistent with Ag capture results that reveal better *M. tuberculosis* control among HIV −ve persons relative to HIV +ve (Figure 2 and Table 2).

Antagonistically, levels of TMKmt Ag detected by PAb-0656 among HIV +ve ATB did not demontrate capability to different ATB from LTBI but were evidently greater than those detected for NTB (OD>0.250) (Figure 3 and Table 3). Specifically, Ab-detection EIAs based on UG-peptide 2 pointed to equally high IgG titres in both LTBI and ATB for HIV +ve and HIV −ve (much as clearly lower IgG titres [OD<1.00] were noted for PLWHA with NTB relative to LTBI and ATB regardless of serostatus). Therefore, TMKmt specific Ab and Ag levels detected by UG-peptide 2 epitope and its derivative PAb-0656 were unable to differentiate ATB from LTBI. Nonetheless, Ab detection based on the same epitope could differentiate NTB from LTBI and ATB regardless of HIV serostatus, a result relevant towards determining who is exposed and instituting TB chemoprophylaxis.

The translational significance of our results lies in Research and Development (R & D) of low cost, easy to use, rapid diagonostic tests for use at the point of care (POC), particularly within resource constrained settings. On one hand, we argue that quantitative RDTs that can designate the different levels of TMKmt Ag and host specific Ab responses based on UG-peptide 1 and its derivative PAb-655 respectively, can be used to easily and rapidly desgnate ATB from LTBI among the TB high burden section of HIV sero-positive persons. On the other hand, similar host specific TMKmt IgG (as has equally been deemed for IgM) [22,23] detecting RDTs based on TMKmt Ab detection using UG-peptide 2, can be explored to designate TB exposure from NTB among either young children in TB endemic settings or adults from TB low burden settings who travelled recently to a TB endemic region. In either case, these RDTs are relevent to the clinician in deciding when to institute TB chemo-therapy and or prophylaxis.

A notable potential limitation to the translational application of UG-peptide 1 for serodiagnosis of ATB, is the potential for cross-reactivity with TMK of other bacteria species other than TMKmt as predicted by basic local sequence alignments tool (BLAST) analysis across the National Center for Biotechnology Information (NCBI) microbial genome database (Supporting file S3) [28]. Specifically, although 100% sequence identity was only observed with TMKmt from various *M. tuberculosis* species, additional protein homology of more than 60% sequence identity was noted with TMK of Corynebacteria spp, Gordonia spp, Tomitella spp, Rhodococcus spp, Segniliparus spp, Dietzila spp, and Tulicella spp. Note, however, that the latter are not documented common pathogens of opportunitic co-infection with HIV/AIDS as the case with *M. tuberculosis* [1, 2]. In addition, the extent of HIV associated B cell immuno-deficiency and use of highly active retroviral therapy (HAART)-will result in variation of readings for antibody EIAs, making antigen capture a mandadtory pararell requirement. These limitations may nevertheless, be over-shadowed by several unfarthomed advantages offered by any hope of TB sero-diagnosis, such as the potential for wide-spead application of this assay towards diagnosis of ATBI among pediatric HIV patients who do not yield sufficient amounts of sputum required for conventiaonl microscopy or culture.

## Conclusion

In conclusion, *M. tuberculosis* growth and proliferation-which actively occurs in the blood of PLWHA due to the dessiminated nature of TB disease seen, is ably and precisely demonstrated by TMKmt Ag capture EIAs based on UG-peptide 1. Moreover, the immunodeficiency present in AIDS is consistently demarcated by a corresponding inversion of host-specific IgG responses. TMKmt Ab and Ag detecting EIAs based on UG-peptide 1 and its derivative PAb can accurately demarcate ATB from LTBI and NTB among HIV +ve subjects. Host specific TMKmt IgG detection using UG-peptide 2, can be explored to designate TB exposure among either young children in TB endemic settings or adults from TB low burden settings who travelled recently to a TB endemic region

## Supplementary Material

Suppl 1

Suppl 2

Suppl 3

## Figures and Tables

**Table 1:** Statistical values for levels of TMKmt Ag detected by UG-peptide 1 derivative PAb-0655 EIAs among 281 serum samples [HIV +ve (139) or HIV −ve (47) ATB versus to LTBI (83) and No TB (10)].

Clinical status of samples	ATB, HIV +ve (n=139)	ATB, HIV −ve (n=47)	LTBI (n=83)	NTB (n=10)	rTMKmt + Control (n=2)
Function (ϝ)	PAb-0655 (UG-Peptide 1 Derivative) Based Direct TMKmt Ag Detection					
**Best fit values**						
Y Intercept	0.8081 ± 0.0329	0.2557 ± 0.0182	0.4577 ± 0.0129	0.2121 ± 0.0213	0.1660 ± 0.0112
Slope	−0.0017 ± 0.0004	0.0006 ± 0.0006	−0.0026 ± 0.0003	0.0019 ± 0.0034	0.9910 ± 0.0071
**Statistical significance**						
P value	<0.0001	0.3513	<0.0001	0.5912	<0.0001
Deviation from 0	Significant	Not Significant	Significant	Not Significant	Significant
**95% Confidence Interval**						
Y Intercept	0.7446 to 0.8715	0.2195 to 0.2918	0.4325 to 0.4829	0.1675 to 0.2567	0.1179 to 0.2141
Slope	−0.0025 to −0.0010	−0.0007 to 0.0019	−0.0031 to −0.0020	−0.0053 to 0.0091	0.9606 to 1.021
**Goodness of fit**						
DFd	1.000, 276.0	1.000, 92.00	1.000, 164.0	1.000, 18.00	1.000, 2.000
R Square	0.0635	0.0095	0.3619	0.0163	0.9999
(n)x2	278	94	166	20	4
Sy.x	0.2686	0.0867	0.0821	0.044	0.0071

**Table 2:** Statistical values for levels of anti-TMKmt specific IgG responses detected by UG-peptide 1 based EIAs among 214 serum samples [HIV +ve (122) or HIV−ve (37) ATB versus LTBI (47) and No TB (8)].

Clinical status of samples	ATB, HIV +ve (n=122)	ATB, HIV −ve (n=37)	LTBI (n=47)	NTB (n=8)
Function (ϝ)	UG-PEPTIDE 1 Based Direct Ant-TMKmt IgG specific Ab Detection			
**Best fit values**				
Y Intercept	1.349 ± 0.0913	2.511 ± 0.2340	0.4543 ± 0.0931	0.5139 ± 0.1684
Slope	0.0006 ± 0.0013	−0.0246 ± 0.0107	0.0073 ± 0.0034	0.0037 ± 0.0334
**Statistical significance**				
P value	0.6665	0.025	0.0334	0.9139
Deviation from 0	Not Significant	Significant	Significant	Not Significant
**95% Confidence Interval**				
Y Intercept	1.170 to 1.528	2.044 to 2.978	0.2690 to 0.6396	0.1527 to 0.8751
Slope	−0.0020 to 0.0031	−0.0460 to −0.0031	0.0006 to 0.0140	−0.0679 to 0.0752
**Goodness of fit**				
DFd	1.000, 242.0	1.000, 72.00	1.000, 92.00	1.000, 14.00
R Square	0.000769	0.06782	0.04824	0.000866
(n)x2	244	74	94	16
Sy.x	0.7087	0.9863	0.4443	0.3056

**Table 3:** Statistical values for levels of TMKmt Ag detected by UG-peptide 2 derivative PAb-0656 EIAs among 281 serum samples [HIV +ve (139) or HIV −ve (47) ATB versus to LTBI (83) and No TB (10)].

Clinical status of samples	ATB, HIV +ve (n=139)	ATB, HIV −ve (n=47)	LTBI (n=83)	NTB (n=10)	rTMKmt + Control (n=2)
Function (ϝ)	PAb-0656 (UG-Peptide 2 Derivative) Based Direct TMKmt Ag Detection				
**Best fit values**					
Y Intercept	0.1039 ± 0.0275	0.2095 ± 0.0110	0.2994 ± 0.0109	0.2206 ± 0.0361	−0.0430 ± 0.0335
Slope	0.0048 ± 0.0003	0.0009 ± 0.0004	−0.0006 ± 0.0002	−0.0049 ± 0.0058	1.203 ± 0.0212
**Statistical significance**					
P value	< 0.0001	0.0261	0.0123	0.4151	0.0003
Deviation from 0	Significant	Significant	Significant	Not Significant	Significant
**95% Confidence Interval**				
Y Intercept	0.0500 to 0.1578	0.1876 to 0.2313	0.2780 to 0.3208	0.1448 to 0.2965	−0.1873 to 0.1013
Slope	0.0041 to 0.0055	0.0001 to 0.0017	−0.0010 to −0.0001	−0.0171 to 0.0074	1.112 to 1.294
**Goodness of fit**					
DFd	1.000, 276.0	1.000, 92.00	1.000, 164.0	1.000, 18.00	1.000, 2.000
R Square	0.4194	0.05262	0.03757	0.03722	0.9994
(n)x2	278	94	166	20	4
Sy.x	0.228	0.0524	0.0697	0.0747	0.0212

**Table 4:** Statistical values for levels of anti-TMKmt specific IgG responses detected by UG-peptide 2 based EIAs among 214 serum samples [HIV +ve (122) or HIV−ve (37) ATB versus LTBI (47) and No TB (8)].

Clinical status of samples	ATB, HIV +ve (n=122)	ATB, HIV −ve (n=37)	LTBI (n=47)	NTB (n=8)
Function (ϝ)	UG-Peptide 2 Based Direct Anti-TMKmt IgG Ab Detection			
**Best fit values**				
Y Intercept	1.303 ± 0.07432	1.091 ± 0.1794	1.029 ± 0.09913	0.9316 ± 0.2348
Slope	0.0012 ± 0.0011	0.0067 ± 0.0082	0.0021 ± 0.0036	−0.0085 ± 0.047
**Statistical significance**				
P value	0.2439	0.4155	0.5664	0.8569
Deviation from 0	Not Significant	Not Significant	Not Significant	Not Significant
**95% Confidence Interval**				
Y Intercept	1.158 to 1.449	0.7330 to 1.449	0.8317 to 1.226	0.4279 to 1.435
Slope	−0.0008 to 0.0034	−0.0097 to 0.0232	−0.0051 to 0.0092	−0.1083 to 0.09122
**Goodness of fit**				
DFd	1.000, 242.0	1.000, 72.00	1.000, 92.00	1.000, 14.00
R Square	0.0056	0.0092	0.0036	0.0024
(n)x2	244	74	94	16
Sy.x	0.5769	0.7561	0.4729	0.4262

## Abbreviations

Ab: Antibody
AFB: Acid Fast Baccilli
Ag: Antigen
OD: Optical Density
EIA: Enzyme Immuno-Assays
ELISA: Enzyme Linked Immuno-Adsorbent Assays
*M. tuberculosis*: *Mycobacterium Tuberculosis*
TB: Tuberculosis
TMKmt: *Mycobacterium Tuberculosis* Thymidylate Kinase
